# A Genotype/Phenotype Study of *KDM5B*-Associated Disorders Suggests a Pathogenic Effect of Dominantly Inherited Missense Variants

**DOI:** 10.3390/genes15081033

**Published:** 2024-08-06

**Authors:** Maria Carla Borroto, Coralie Michaud, Chloé Hudon, Pankaj B. Agrawal, Katherine Agre, Carolyn D. Applegate, Alan H. Beggs, Hans T. Bjornsson, Bert Callewaert, Mei-Jan Chen, Cynthia Curry, Orrin Devinsky, Tracy Dudding-Byth, Kelly Fagan, Candice R. Finnila, Ralitza Gavrilova, Casie A. Genetti, Susan M. Hiatt, Friedhelm Hildebrandt, Monica H. Wojcik, Tjitske Kleefstra, Caroline M. Kolvenbach, Bruce R. Korf, Paul Kruszka, Hong Li, Jessica Litwin, Julien Marcadier, Konrad Platzer, Patrick R. Blackburn, Margot R. F. Reijnders, Heiko Reutter, Ina Schanze, Joseph T. Shieh, Cathy A. Stevens, Zaheer Valivullah, Marie-José van den Boogaard, Eric W. Klee, Philippe M. Campeau

**Affiliations:** 1 Centre de Recherche Azrieli du CHU Sainte-Justine, University of Montreal, Montreal, QC H3T 1C5, Canadachloehudon@outlook.fr (C.H.); 2 The Manton Center for Orphan Disease Research, Divisions of Newborn Medicine and of Genetics and Genomics, Boston Children’s Hospital, Harvard Medical School, Boston, MA 02115, USA; 3 Department of Clinical Genomics, Mayo Clinic, Rochester, MN 55902, USA; 4 Department of Genetic Medicine, McKusick-Nathans Institute, Johns Hopkins University School of Medicine, Baltimore, MD 21205, USA; cdapplegate@jhmi.edu (C.D.A.);; 5 The Manton Center for Orphan Disease Research, Division of Genetics and Genomics, Boston Children’s Hospital, Harvard Medical School, Boston, MA 02115, USA; beggs@enders.tch.harvard.edu (A.H.B.);; 6 Louma G. Laboratory of Epigenetic Research, Faculty of Medicine, University of Iceland, 101 Reykjavik, Iceland; 7 Department of Genetics and Molecular Medicine, Landspitali University Hospital, 101 Reykjavik, Iceland; 8 Center for Medical Genetics, Ghent University Hospital, 9000 Ghent, Belgium; 9 Department of Genetics, University of Alabama at Birmingham, Birmingham, AL 35294, USA; 10 Genetic Medicine, University of California San Francisco/Fresno, Fresno, CA 93701, USA; 11 Departments of Neurology, Neuroscience, Neurosurgery and Psychiatry, NYU School of Medicine, New York, NY 10016, USA; 12 Hunter Genetics, Newcastle, NSW 2298, Australia; 13 UCSF Benioff Children’s Hospital, San Francisco, CA 93940, USA; 14 HudsonAlpha Institute for Biotechnology, 601 Genome Way, Huntsville, AL 35806, USA; 15 Department of Neurology, Mayo Clinic, Rochester, MN 55902, USA; 16 Department of Pediatrics, Boston Children’s Hospital, Harvard Medical School, Boston, MA 02115, USA; 17 Department of Human Genetics, Radboud University Medical Center, 6525 GA Nijmegen, The Netherlands; 18 Institute of Anatomy and Cell Biology, Medical Faculty, University of Bonn, 53127 Bonn, Germany; 19 GeneDx LLC, Gaithersburg, MD 20877, USA; 20 Department of Human Genetics, School of Medicine, Emory University, Atlanta, GA 30322, USA; 21 Department of Neurology, University of California, San Francisco Benioff Children’s Hospital, San Francisco, CA 94158, USA; 22 Division of Medical Genetics, Alberta Children’s Hospital, Calgary, AB T3B 6A8, Canada; 23 Institute of Human Genetics, University of Leipzig Medical Center, 04103 Leipzig, Germany; 24 Department of Pathology, St. Jude Children’s Hospital, Memphis, TN 38105, USA; 25 Department of Human Genetics, Radboud Institute for Molecular Life Sciences, Radboud University Medical Center, 6525 GA Nijmegen, The Netherlands; 26 Institute of Human Genetics, University Hospital of Bonn, 53127 Bonn, Germany; 27 Institute of Human Genetics, 39120 Magdeburg, Germany; 28 Division of Medical Genetics, Department of Pediatrics, University of California, San Francisco Benioff Childen’s Hospital, San Francisco, CA 94143, USA; 29 Department of Pediatrics, University of Tennessee College of Medicine, Chattanooga, TN 38103, USA; 30 Center for Mendelian Genomics, Broad Institute Harvard, Cambridge, MA 02142, USA; 31 Department of Genetics, University Medical Centre Utrecht, P.O. Box 85500, 3508 GA Utrecht, The Netherlands; 32 Department of Quantitative Health Sciences, Mayo Clinic, Rochester, MN 55902, USA

**Keywords:** neurodevelopmental disorders, intellectual disabilities, histone demethylation, KDM5, epigenetics, genetic syndromes, polygenetic interactions

## Abstract

Bi-allelic disruptive variants (nonsense, frameshift, and splicing variants) in *KDM5B* have been identified as causative for autosomal recessive intellectual developmental disorder type 65. In contrast, dominant variants, usually disruptive as well, have been more difficult to implicate in a specific phenotype, since some of them have been found in unaffected controls or relatives. Here, we describe individuals with likely pathogenic variants in *KDM5B*, including eight individuals with dominant missense variants. This study is a retrospective case series of 21 individuals with variants in *KDM5B*. We performed deep phenotyping and collected the clinical information and molecular data of these individuals’ family members. We compared the phenotypes according to variant type and to those previously described in the literature. The most common features were developmental delay, impaired intellectual development, behavioral problems, autistic behaviors, sleep disorders, facial dysmorphism, and overgrowth. DD, ASD behaviors, and sleep disorders were more common in individuals with dominant disruptive *KDM5B* variants, while individuals with dominant missense variants presented more frequently with renal and skin anomalies. This study extends our understanding of the *KDM5B*-related neurodevelopmental disorder and suggests the pathogenicity of certain dominant *KDM5B* missense variants.

## 1. Introduction

The nucleosomal packaging system enables DNA to be densely arranged inside cellular nuclei while maintaining its accessibility and capacity for modulation [1]. A nucleosome is an octamer of histone proteins that can be wrapped by close to 150 base pairs. Gene accessibility and, therefore, transcription can be altered in accordance with how tightly the genomic region interacts with histones [2,3]. These interactions are dynamically manipulated through epigenetic mechanisms, like chemical modifications [4]. The evolutionarily conserved methylation of various lysine residues in histones has been well characterized, as have several mediating enzymes [4,5]. Twenty-seven lysine methylase (KMT)- and 24 lysine demethylase (KDM)-encoding genes have been described. Heterozygous variants in seven KMT and seven KDM genes have been associated with developmental disorders [6].

The fourth lysine residue of histonine H3 (H3K4) appears to be the most extensively targeted histone methylation site, while mutations in 10 of its epigenetic regulators (KMT2A, KMT2C, KMT2D, KMT2F, KDM1A, KDM5A, KDM5B, KDM5C, PHF21A, and PHF8) have been associated with neurodevelopmental disorders (NDDs) [7]. De novo variants in *PHF21A* (MIM: 608325) cause NDDs with behavioral abnormalities, craniofacial dysmorphism, overgrowth, and potential epilepsy [8]. *PHF8* (MIM: 300560) variants cause a spectrum of X-linked NDDs and facial dysmorphism [9]. De novo variants in *KDM1A* (MIM: 609132) cause developmental delay (DD) with distinctive facial dysmorphisms. Sixteen mutant KMT2 isoforms can lead to a variety of syndromic NDDs (Wiedemann–Steiner syndrome, Coffin–Siris syndrome, Kabuki syndrome, Cornelia De Lange syndrome, and Rubinstein–Taybi syndrome) [10]. 

Regarding the KDM5 family, variants in *KDM5C* (MIM: 314690) cause X-linked intellectual disability (ID) [11,12], and female carriers may present with mild ID [13]. *KDM5A* (MIM: 180202) variants were initially associated with autosomal recessive ID and facial dysmorphisms [14] and posteriorly identified in an NDD cohort that included de novo variants. Probands also presented with autism spectrum disorder (ASD) and a lack of speech [15]. Large-scale exome studies have confirmed that *KDM5B* is also associated with cognitive function [16]. Variants in this gene have been identified in NDD and ASD studies [17,18,19], and *KDM5B* has been identified as a risk gene for de novo variants leading to primary complex motor stereotypies [20].

In the deciphering developmental disorders (DDD) cohort, *KDM5B* was the only gene enriched for both biallelic variants and de novo mutations (DNMs), the latter including three missense and six loss-of-function (LoF) variants. Three individuals with bi-allelic LoF variants and one individual compound heterozygous for a splice site variant and a large deletion were diagnosed with autosomal recessive intellectual developmental disorder-65 (MRT65) (OMIM: 618109) [17,18]. Interestingly, dominant LoF variants showed non-segregation within families. Likewise, in a different ASD study, the same DNMs were seen in ASD patients and in unaffected siblings [18]. These findings suggest that the *KDM5B*-related NDD follows an unusual pattern and requires further exploration, especially in terms of genotype–phenotype associations.

Given the knowledge gaps regarding *KDM5B* heterozygous variants and the *KDM5B*-associated NDD at large, we established a cohort with 21 new cases and studied the clinical and genetic presentations, as well as the genotype–phenotype associations.

## 2. Methods

Twenty-one individuals from unrelated families with *KDM5B* variants (GenBank: NM_001314042.1; MIM: 605393) were included in this case series. Individuals were identified among cohorts of collaborating investigators by the web-based tool GeneMatcher, which allows clinicians and researchers interested in the same gene to find and communicate with each other [21]. Exome sequencing, performed on both research and clinical bases according to protocols laid out by the respective laboratories, led to identification of 18 novel variants and 4 bi-allelic variants of *KDM5B* in 21 individuals. Phenotypic data were recorded by clinicians. Informed consent was obtained from all subjects involved in the study, which was approved by the Ethics Committee of CHU Sainte-Justine. Written consent was obtained for photographs. Clinical information was shared according to local institutional review boards. 

## 3. Results

### 3.1. Genotypic Findings in New Cases of KDM5B-Related Disorder

The clinical phenotypes of 21 probands with variants in *KDM5B* are outlined in Table S1. Nineteen individuals harbored a dominant variant, while two individuals were compound heterozygous. Three of the four alleles were inherited in their case from unaffected parents. Among the individuals with dominant variants, nine individuals were female, and ten were male. Among the individuals with bi-allelic variants, one was female, and one was male. The age at last evaluation ranged from 2 days of life to 20 years for the individuals with dominant variants. The individuals with bi-allelic variants were each aged 4 and 28 years old. Table 1 shows the genotypical data of our cohort, while detailed ACMG classifications can be found in Table S2. 

**Figure 1:**
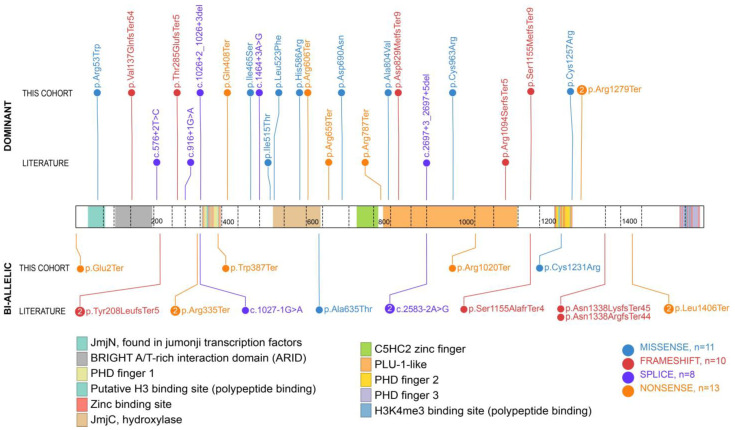
Dominant and bi-allelic *KDM5B* variants from this cohort and the literature represented within the KDM5B protein. The active regions are named and highlighted in different colors according to the legend. Variant types are also indicated, and if the same one was shared by multiple individuals, it is shown as a circle with the total number. Illustration is adapted from https://proteinpaint.stjude.org/.

Overall, 22 variants in *KDM5B* were identified in these 21 individuals (Table 1, Figure 1). Two had apparently bi-allelic *KDM5B* variants: one being compound heterozygous for two nonsense variants and the other carrying one inherited missense variant and one de novo nonsense variant for which phasing could not be determined. The eighteen dominant variants included eight missense variants, three nonsense variants, two splice site variants, four frameshift variants, and one was a whole gene deletion. Of the frameshift variants, three were a result of deletions and one was a result of an insertion. Only two dominant variants were inherited from affected parents (individuals No. 15 and No. 18), while the others were confirmed to be de novo.

Two individuals (Nos. 10 and 11) shared a dominant nonsense *KDM5B* variant (c.3835C>T, p.Arg1279Ter), further suggesting a deleterious nature for this variant. Both patients presented with an auditory processing disorder.

An amino acid conservation analysis demonstrates that most affected positions are highly conserved in vertebrates (Figure S1). The nucleotide of the splice variant in individual No. 13 was well conserved among other vertebrate species whereas that of individual 14 No. was not.

Individual No. 19 had a large deletion affecting several other genes (chr1:g.202320001_203070000del), which could have certainly compounded the phenotype. 

Regarding the nine missense variants, they were all conserved in other vertebrates except for those found in individuals No. 2 and 21. Regarding their conservation in other KDM5 subfamily members, they were well conserved, except for in individuals Nos. 2, 3, 5, and 21. However, note that the amino acid substituted in individual No. 3, p.Asp690, is a glutamate in KDM5A/C/D, which is an acidic residue like aspartate, while the variant in individual No. 3 has asparagin rather than aspartate, which is a polar, non-acidic residue. 

Regarding the splicing variants, for individual No. 13 (c.1026+2_1026+3del), the Pangolin model predicts a frameshift variant, which we classified as likely pathogenic (see Table S2). The variant found in individual No. 14 (c.1464+3A>G) was classified as a variant of unknown significance (VUS). It is predicted to be splice donor region variant (with a Pangolin score of 0.36).

### 3.2. Clinical Findings in New Cases of KDM5B-Related Disorder

Data on their neurodevelopment were available for 13 individuals with dominant variants, of which one presented with isolated motor delay, and over half (7/13) presented with speech delay, including one proband who was non-verbal. Overall, close to 80% of individuals with dominant variants presented with an ID or DD, and 40% had a global DD (GDD). Both individuals with bi-allelic variants presented with a mild ID, and one of them presented with a GDD. ID (assessed after 5 years of age) was observed in eight of the fifteen individuals with dominant variants. One of them had mild, four had moderate, and three had severe IDs. These findings suggest that *KDM5B* variants, whether dominant or bi-allelic, can impair neurodevelopment and cognition. They might also suggest that cognition is more heavily impaired in bi-allelic variants.

Autistic behaviors were noted in half of individuals with dominant variants (10/19), out of which, four did not display concomitant intellectual disability. Behavioral problems (8/19), such as ADHD (4/8), were common among individuals with dominant variants. Anxiety, aggressivity, and a low frustration threshold were also reported. Both individuals with bi-allelic variants displayed behavioral problems such as mood swings and aggressivity, but they did not display autistic behaviors. These findings suggest that behavioral abnormalities are a prominent finding in the *KDM5B*-related disorder, while dominant variants are also linked to ASD.

Three individuals with dominant variants presented with macrocephaly, meaning an occipital frontal circumference (OFC) above two standard deviations (SDs) or the 98th percentile. Both individuals with bi-allelic variants presented with macrocephaly. Two other individuals presented with an OFC above the 90th percentile. The majority of the patients with dominant variants (9/15) and one patient with bi-allelic variants had weights above the 90th percentile. Over a third of the patients with dominant variants (6/15) and one patient with bi-allelic variants had heights above the 90th percentile. In contrast, only one individual presented with abnormally small OFC, height, and weight measurements (<−2 SDs), and isolated microcephaly was seen in two other individuals. Interestingly, one individual (No. 19) was born with normal measurements, but, by the beginning of adulthood, had gigantism, macrocephaly, and severe obesity. Overall, these findings suggest that overgrowth might be a prominent finding in the *KDM5B*-related disorder.

Structural brain malformations were noted for three of the seven individuals with dominant variants and one individual with bi-allelic variants. Anomalies included polymicrogyria, enlarged extra-axial spaces or ventricles, the agenesis of the corpus callosum, and delayed myelination. These findings suggest that brain imaging might be abnormal for a portion of individuals with *KDM5B*-related disorder, although not for the majority.

**Figure 2:**
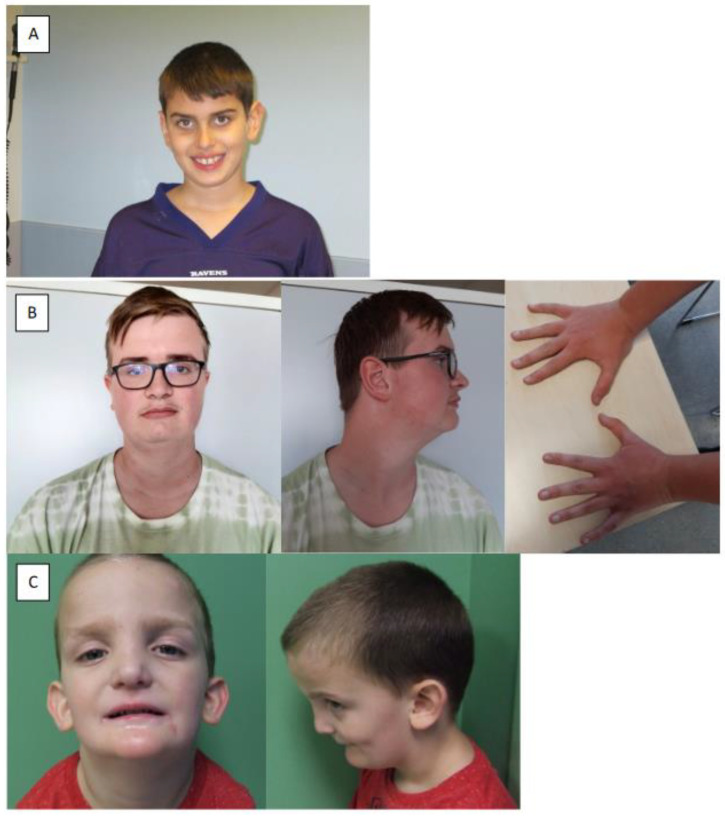
Photographs of individuals with *KDM5B* variants. (**A**) Individual No. 5 (p.Ala804Val) at the age of 10 years old. Note the macrotia, straight eyebrows, telecanthus, the full nasal tip, and the broad mouth. (**B**) Individual No. 19 (Whole gene deletion) at the age of 18 years old. Note the brachycephaly, elongated face, high forehead, temporally sparse hair, broad eyebrows, bilaterally temporal narrowing, downslanted palpebral fissures, short philtrum, facial asymmetry (left side is slightly shorter), increased cervical length and width, and large hands with long, tapered fingers. (**C**) Individual No. 21 (p.Glu2Ter and p.Trp387Ter) at the age of 4 years old. Note the broad forehead, the midface retraction, the full nasal tip, and the micrognathia.

Facial dysmorphisms were common in individuals with dominant variants (13/19). Photographs of three individuals are shown in Figure 2. A broad, high, or prominent forehead (4/13), synophrys (4/13), low eyebrows (3/13), full nasal tip (3/13), and prominent large ears (3/13) were frequently reported. Both individuals with bi-allelic variants presented with facial dysmorphisms, including a broad forehead and full nasal tip. Joint hypermobility was present in 4/19 of the individuals with dominant variants. Four individuals had atrophic hands including two with a Swan neck deformity. One individual with the bi-allelic variant presented with joint hypermobility and atrophic hands, including a Swan neck deformity. These findings suggest that facial and hand dysmorphisms could be a recurrent finding in the *KDM5B*-related disorder.

Sleeping disorders were common among the individuals with dominant variants, (9/19) with two of the nine individuals presenting with obstructive sleep apnea. Sleep issues were not noted in the individuals with bi-allelic variants. These findings suggest that sleeping disorders are a common comorbidity in the *KDM5B*-related disorder, specifically in individuals with dominant variants. 

Cardiac anomalies were only noted in individuals with dominant variants as well. Individual No. 17 underwent surgery for an atrial septal defect (ASD) at 3 years of age. Individual 6 also presented with an ASD, as well as a right aortic arch with an aberrant left subclavian artery and left ligamentum; a muscular ventricular septal defect (VSD); and a dilated/hypertensive right ventricle. Additionally, individual 15 presented with a vascular malformation. These findings suggest that nonspecific cardiovascular anomalies might be concomitant with the *KDM5B*-related disorder, especially in individuals with dominant variants. 

Renal and dermatologic anomalies were also found only in these individuals. Renal anomalies included chronic kidney disease, hydronephrosis, and ureteral dilatation. The skin anomalies included keratosis pilaris, ulerythema ophryogenes, and hypertrichosis. Overall, these findings might suggest that extra-neurological organ anomalies, including cardiac, renal, and dermatologic anomalies, are commonly found in the *KDM5B*-related disorder, especially in individuals with dominant variants. 

### 3.3. Comparison of Our Findings with the Literature

Table 2A,B compare the clinical findings of this study with those already described in the literature (see Table S3 for a detailed explanation of the cases retained from the literature). Overall, the six previously reported cases with dominant *KDM5B* variants resemble those in our cohort. However, our study is the first to report concomitant ADHD, dermatologic anomalies, cardiac anomalies, sleep disorders, and joint hypermobility. Additionally, our findings suggest that autistic behaviors are more common than previously known.

Previously reported cases of bi-allelic *KDM5B* variants have reported that ASD, ADHD, sleep disorders, and cardiac anomalies could also be seen. Their absence in the two individuals included in our cohort seems to confirm that such clinical features are found in a minority of individuals with bi-allelic *KDM5B* variants. Interestingly, this study is the first to describe behavioral abnormalities in individuals with bi-allelic *KDM5B* variants. 

### 3.4. Comparison of Phenotypes Based on Variant Type

Table 2C compares the dominant missense and dominant disruptive variants (nonsense, frameshift, and splicing) included in our cohort. The extra-neurological findings were strikingly similar, with the exception of renal and skin anomalies. Renal malformations were four times more common in individuals with disruptive variants (38% vs. 9%). Over a third of this group had skin anomalies (38% vs. 0%), but dermatologic anomalies were not noted in individuals with missense variants. These findings might suggest that a distinguishable finding between disruptive and missense *KDM5B* dominant variant-associated phenotypes is that there is skin involvement in the latter, while renal anomalies are also considerably more linked to missense *KDM5B* variants.

In terms of neurological phenotype, missense and disruptive variants were linked to slightly different presentations. DDs were less than half as commonly linked to missense variants than to disruptive variants (38% vs. 91%), while IDs were more noted (50% vs. 36%). ASD was more commonly linked to disruptive varients (64% vs. 38%). Overall, these findings might suggest that *KDM5B* LoF variants impair neurodevelopment more heavily than *KDM5B* missense variants, although the latter can impair cognition as much as disruptive variants, if not slightly more.

## 4. Discussion

The present report describes 21 new individuals with rare *KDM5B* variants, including 18 new cases with dominant *KDM5B* variants. The most common features in individuals with *KDM5B* variants are neurodevelopmental delay, ID, behavioral problems, autistic behaviors, sleeping disorders, facial dysmorphology, and overgrowth. Individuals with dominant disruptive (nonsense, frameshift, and splicing) *KDM5B* variants were characterized more commonly by DD, ASD behaviors, and sleep disorders. In addition, individuals with dominant missense variants presented more frequently with renal and skin anomalies. 

When comparing individuals with dominant variants in our study with those described in the literature (Table 2) [18,22], only a few major dissimilarities arise that may relate to an ascertainment bias. The individuals in our study more frequently showed sleep disorders (47% vs. 0%) and joint hypermobility (21% vs. 0%). Other neurological features, such as seizures, sensorineural hearing impairment, and white matter hypodensities or cerebellar vermis hypoplasia on imaging, were more common in the literature (83% vs. 26%). 

Both individuals with bi-allelic variants in our study presented with ID, facial dysmorphism, and behavioral problems, but not ASD. We previously reported an association of autistic behaviors with mono-allelic rather than bi-allelic variants for another epigenetic regulator, *ACTL6B* [23]. Our findings suggest that a similar phenomenon occurs in the *KDM5B*-associated disorder. Additionally, despite *KDM5B* being associated with stereotypies, these were only explicitly noted in individual No. 14. However, stereotypies could have been underreported as these are often classified under the umbrella term of “autistic behaviors” [20]. Finally, seizures were absent in bi-allelic as well as in dominant *KDM5B* variants in our cohort. 

Four cases of bi-allelic *KDM5B* variants with intellectual developmental disorder autosomal recessive-65 (MRT65) (OMIM: 618109) have been described in the literature [17,18]. The phenotype of the individuals with biallelic *KDM5B* variants in our study are largely comparable to those previously reported (Table 2) [17,18,24]. Moderate-to-severe DD and impaired intellectual development were common to all. 

We found that, in our cohort, when assessed, there was a disproportionately high number of patients with macrocephaly or borderline macrocephaly (6/13), obesity (10/16), and heights above the 90th percentile (7/17). These findings lead us to suggest that overgrowth might be a common clinical feature of the *KDM5B*-related disorder, as it is for the NDD associated with *PHF21A*, another gene involved in H3K4 methylation [8].

The nucleosome remodeling and deacetylase (NuRD) complex regulates chromatin structure. It is composed of multiple enzymes and links histone deacetylase activity with ATP-dependent nucleosome remodeling activity. As such, it functions closely with *KDM5B* in chromatin remodeling [25]. Variants in subunits of NuRD, such as chromodomain helicase DNA-binding protein 3 and 4 (*CHD3* and *CHD4*), are linked to dominant neurodevelopmental syndromes. Both entities share characteristics with the *KDM5B*-related disorder such as ID, macrocephaly, and craniofacial characteristics including a broad prominent forehead, frontal bossing, ocular hypertelorism, and a wide nasal bridge [26]. However, individuals with *CHD4* variants have a high prevalence of congenital heart defects [27], while only 3/21 individuals in this cohort with *KDM5B* variants presented with cardiovascular anomalies. 

Unlike individuals with *KDM5B* variants, individuals with *KDM5C* variants frequently present with profound verbal deficiency, short stature, spasticity, and microcephaly. Aggressive behavior, dysmorphic features, and strabismus are also common [11], as in individuals with *KDM5B* variants. Individuals with *KDM5A* variants in the literature present more frequently with ASD than individuals with *KDM5B* variants in this study. A lack of speech is more specific to individuals with *KDM5A* variants [15]. Our study contributes to the recognition of the crucial role that H3K4 demethylation plays in neurodevelopment.

Martin et al. reported an individual with a recessive case who had ADHD, experienced sleep disturbances, and had generalized joint laxity. These characteristics are similar to those of several dominant cases. Mangano et al. described a patient with a frameshift *KDM5B* variant, two de novo variants, and a 2q deletion of 8.2 Mb, presenting with facial and finger dysmorphism, severe intellectual and motor disorders, and a rare epileptic syndrome. They proposed that the dysmorphic features were secondary to a *KDM5B* variant [28]. However, only one of the three patients with frameshift variants in our cohort presented with dysmorphic features, which contrasts with the patient presented by Mangano et al. In our opinion, there is a spectrum of recessiveness in *KDM5B*-related diseases [29]. 

Martin et al. proposed that heterozygous LoF variants could be pathogenic with incomplete penetrance [18]. Lebrun et al. suggested two additional hypotheses: autosomal monoallelic expression leading to allele-biased expression; or gain-of-function (GoF) alterations leading to the loss of the ARID domain in a new, deleterious KDM5B protein [22]. There are 40 LoF variants listed in GnomAD resulting in an observed/expected ratio of 0.57 for LoF variants (GnomAD non neuro v2.1.2). This is in line with a partial penetrance of heterozygous LoF *KDM5B* variants. 

Further functional studies will be required in order to elucidate the underlying mechanisms of this unconventional NDD. Additionally, despite the crucial role of KDM5B in epigenetic demethylation, methylation signatures have not yet emerged for dominant or bi-allelic variants in *KDM5B* [18]. An analysis of a larger cohort including de novo missense variants would be interesting to determine if a robust and statistically significant methylation signature can emerge. 

## 5. Conclusions

In conclusion, our study extends the understanding of the *KDM5B*-related NDD and suggest the pathogenicity of certain dominant missense *KDM5B* variants. Recurrent clinical findings in our cohort included DD, ID, behavioral abnormalities, autistic behaviors, sleeping disorders, renal and skin anomalies, facial dysmorphology, and potential overgrowth. DD, ASD behaviors, and sleep disorders were more common in individuals with dominant disruptive *KDM5B* variants, while individuals with dominant missense variants presented more frequently with renal and skin anomalies. Genotypic interpretation remains difficult in the light of reduced penetrance. Future epigenetic studies might uncover episignatures which may help differentiate pathogenic from benign variants.

## Figures and Tables

**Table 1:** *KDM5B* variants identified in the 21 individuals of our cohort.

No.	Genetic Variant (hg19/GRCh37)	cDNA Variant (NM_001314042.1)	Protein Variant	Inheritance	Predicted Effect	Pathogenicity Score (CADD) ^a^	gnomAD (Allele Count) ^b^
1	chr1:g.207207771A>G	c.3769T>C	p.Cys1257Arg	De novo	Missense	30	N/A
2	chr1:g.20716061A>G	c.2887T>C	p.Cys963Arg	De novo	Missense	22.2	N/A
3	chr1:g.202718129C>T	c.2068G>A	p.Asp690Asn	De novo	Missense	25.8	N/A
4	chr1:g.202722085T>C	c.1757A>G	p.His586Arg	De novo	Missense	26	N/A
5	chr1:g.202715006G>A	c.2411T>C	p.Ala804Val	De novo	Missense	27.8	N/A
6	chr1:g.202772777T>G	c.157C>T	p.Arg53Trp	De novo	Missense	28.3	N/A
7	chr1:g.202725556A>C	c.1394T>G	p.Ile465Ser	De novo	Missense	29.1	N/A
8	chr1:g.202724476C>G	c.1569C>G	p.Leu523Phe	De novo	Missense	23.9	N/A
9	chr1:g.202719900G>A	c.1816C>T	p.Arg606Ter	De novo	Nonsense	36	2
10	chr1:g.202702711G>A	c.3835C>T	p.Arg1279Ter	Mother negative, father not tested	Nonsense	39	1
11	chr1:g.202702711G>A	c.3835C>T	p.Arg1279Ter	De novo	Nonsense	39	1
12	chr1:g.202727602G>A	c.1222C>T	p.Gln408Ter	De novo	Nonsense	38	N/A
13	chr1:202731824:CACA:CA Deletion (2bp)a	c.1026+2_1026+3del	N/A	De novo	Splicing	N/A	N/A
14	chr1:g.202725483T>C	c.1464+3A>G	N/A	De novo	Splicing	23.2	N/A
15	chr1:g.202711876TTGTC>T	c.2485_2488del	p.Asp829MetfsTer9	Inherited from affected father	Frameshift	N/A	N/A
16	chr1:g.202733231_202733240del	c.853_862del	p.Thr285GlufsTer5	De novo	Frameshift	N/A	N/A
17	chr1:g.202704625insA	c.3463_3464insT	p.Ser1155MetfsTer9	De novo	Frameshift	N/A	N/A
18	chr1:g.202742411AACTdel	c.408_411del	p.Val137GlnfsTer54	Inherited from affected mother	Frameshift	N/A	N/A
19	chr1:g.202320001_203070000del	N/A	N/A	De novo	Whole gene deletion	N/A	N/A
20	chr1:g.202709936G>A	c.3058C>T	p.Arg1020Ter	De novo	Nonsense	44	2
	chr1:g.202702855A>G	c.3691T>C	p.Cys1231Arg	Inherited	Missense	29.3	N/A
21	chr1:g.202777430C>A	c.4G>T	p.Glu2Ter	Inherited from unaffected parents	Nonsense	37	N/A
	chr1:g.202729568C>T	c.1160G>A	p.Trp387Ter		Nonsense	41	N/A

All patients were heterozygous for *KDM5B* variants; ^a^ CADD v1.4 scores range from 1 to 99, with a higher score indicating greater deleteriousness. ^b^ Refers to total allele counts and total frequencies in gnomAD dataset v2.1.1. (allele count is the same in the non-neuro subset). “N/A” indicates “non applicable”.

**Table 2:** Phenotype comparisons.

**(A) Clinical Findings among Individuals with Dominant *KDM5B* Variants in this Study vs. in the Literature**		
**Clinical Finding**	**This Study (n = 19)**	**Literature (n = 6)**
ID	42%	50%
DD	68%	83%
Either ID or DD	79%	83%
Autistic behaviors	53%	33%
Behavioral abnormalities	37%	33%
ADHD	16%	0%
Other neurological findings	26%	83%
Renal anomalies	21%	17%
Skin anomalies	16%	0%
Finger anomalies	26%	17%
Facial dysmorphisms	68%	50%
Sleep disorder	47%	0%
Joint hypermobility	21%	0%
Ophthalmologic anomalies	16%	17%
Cardiac anomalies	11%	0%
**(B) Clinical Findings among Individuals with Bi-Allelic *KDM5B* Variants in This Study vs. in the Literature**		
**Clinical Finding**	**This Study (n = 2)**	**Literature (n = 8)**
ID	100%	75%
DD	50%	100%
Either ID or DD	100%	100%
Autistic behaviors	0%	13%
Behavioral abnormalities	100%	0%
ADHD	0%	13%
Other neurological findings	50%	50%
Renal anomalies	0%	0%
Skin anomalies	0%	0%
Finger anomalies	50%	38%
Facial dysmorphisms	100%	88%
Sleep disorder	0%	13%
Joint hypermobility	50%	13%
Ophthalmologic anomalies	50%	38%
Cardiac anomalies	0%	25%
**(C) Clinical Findings among Individuals with Missense vs. Disruptive Dominant *KDM5B* Variants in This Study**		
**Clinical Finding**	**Individuals with** **Missense Variants (n = 8)**	**Individuals with** **Disruptive Variants** **(n = 11)**
ID	50%	36%
DD	38%	91%
Either ID or DD	63%	91%
Autistic behaviors	38%	64%
Behavioral abnormalities	38%	36%
ADHD	13%	18%
Other neurological findings	25%	27%
Renal anomalies	38%	9%
Skin anomalies	38%	0%
Finger anomalies	25%	27%
Facial dysmorphisms	63%	73%
Sleep disorder	38%	55%
Joint hypermobility	25%	18%
Ophthalmologic anomalies	13%	18%
Cardiac anomalies	13%	9%

## Data Availability

The original contributions presented in the study are included in the article/Supplementary Materials; further inquiries can be directed to the corresponding author.

## Supplementary Materials

The following supporting information can be downloaded at: https://www.mdpi.com/article/10.3390/genes15081033/s1. Figure S1: Amino acids conservation in vertebrates; Table S1: Phenotypic characteristics of individuals with *KDM5B* variants; Table S2: *KDM5B* variants ACMG classification; Table S3: Detailed explanation of the cases retained from the literature.
