# Supplementary material for: A Genotype/Phenotype Study of KDM5B-Associated Disorders Suggests a Pathogenic Effect of Dominantly Inherited Missense Variants
Source: Genes (Basel). 2024 Aug 6;15(8):1033. doi: 10.3390/genes15081033 (PMC11353349; doi:10.3390/genes15081033)
Supplement: Supplementary file 1 [file genes-15-01033-s001.zip › genes-3084693-supplementary.pdf]

Figure S1. Amino acids conservation in vertebrates

**Individual No. 1**					**p.Cys1257**				
Human	L	C	P	H	**C**	R	R	S	E
Rhesus	L	C	P	H	**C**	R	R	S	E
Mouse	L	C	P	H	**C**	R	R	S	E
Dog	L	C	P	H	**C**	R	R	S	E
Elephant	L	C	P	H	**C**	R	R	S	E
Chicken	L	C	P	Q	**C**	R	R	S	E
Xenopus	L	C	P	Q	**C**	H	R	S	E
Zebrafish	L	C	P	L	**C**	L	R	S	T
Human *KDM5A*	L	C	P	L	**C**	M	R	S	R
Human *KDM5C*	L	C	P	L	**C**	M	R	S	R
Human *KDM5D*	L	C	P	L	**C**	M	R	S	R

**Individual No. 2**					**p.Cys963**				
Human	V	Q	Q	A	**C**	L	D	P	S
Rhesus	V	Q	Q	A	**C**	L	D	P	S
Mouse	V	Q	Q	A	**C**	L	D	S	S
Dog	V	Q	Q	A	**C**	L	D	P	S
Elephant	V	Q	Q	A	**C**	L	D	P	N
Chicken	V	Q	M	A	**S**	A	E	Q	N
Xenopus	Q	H	Q	A	**C**	K	D	P	R
Zebrafish	V	H	Q	A	**E**	D	H	P	C
Human *KDM5A*	V	R	L	T	**L**	S	D	P	Q
Human *KDM5C*	V	K	R	T	**L**	A	P	S	A
Human *KDM5D*	V	K	Q	A	**L**	A	P	S	A

**Individual No. 3**					**p.Asp690**				
Human	T	V	Q	K	**D**	M	A	I	M
Rhesus	T	V	Q	K	**D**	M	A	I	M
Mouse	T	V	Q	K	**D**	M	A	I	M
Dog	T	V	Q	K	**D**	M	A	I	M
Elephant	T	V	Q	K	**D**	M	A	I	M
Chicken	T	V	Q	K	**D**	M	A	I	M
Xenopus	S	V	Q	K	**D**	M	A	S	M
Zebrafish	A	V	Q	K	**D**	M	Q	L	M
Human *KDM5A*	M	V	C	K	**E**	L	T	L	M
Human *KDM5C*	A	V	H	K	**E**	M	F	I	M
Human *KDM5D*	A	V	H	K	**E**	M	F	I	M

**Individual No. 4**					**p.His586**				
Human	P	D	L	L	**H**	Q	L	V	T
Rhesus	P	D	L	L	**H**	Q	L	V	T
Mouse	P	D	L	L	**H**	Q	L	V	T
Dog	P	D	L	L	**H**	Q	L	V	T
Elephant	P	D	L	L	**H**	Q	L	V	T
Chicken	P	D	L	L	**H**	Q	L	V	T
Xenopus	P	D	L	L	**H**	Q	L	V	T
Zebrafish	P	D	L	L	**H**	Q	L	V	T
Human *KDM5A*	P	D	L	L	**H**	Q	L	V	T
Human *KDM5C*	P	D	L	L	**H**	Q	L	V	T
Human *KDM5D*	P	D	L	L	**H**	Q	L	V	T

**Individual No. 5**					**p.Ala804**				
Human	E	A	L	E	**A**	K	I	N	K
Rhesus	E	A	L	E	**A**	K	I	N	K
Mouse	E	A	L	E	**A**	K	I	N	K
Dog	E	A	L	E	**A**	K	I	N	K
Elephant	E	A	L	E	**A**	K	I	N	K
Chicken	E	A	L	E	**A**	K	I	S	N
Xenopus	D	A	-	-	**-**	K	L	H	N
Zebrafish	E	V	M	E	**A**	K	L	D	K
Human *KDM5A*	E	A	L	S	**A**	N	F	N	H
Human *KDM5C*	V	A	L	E	**V**	E	D	G	R
Human *KDM5D*	V	A	L	E	**V**	E	D	G	R

**Individual No. 6**					**p.Arg53**				
Human	I	H	K	I	**R**	P	I	A	E
Rhesus	I	H	K	I	**R**	P	I	A	E
Mouse	I	H	K	I	**R**	P	I	A	E
Dog	-	-	R	A	**R**	E	L	R	R
Elephant	-	-	-	-	**-**	-	-	-	-
Chicken	I	G	R	I	**R**	G	L	A	E
Xenopus	I	*N*	K	I	**R**	P	I	A	E
Zebrafish	I	*N*	K	I	**R**	P	I	A	E
Human *KDM5A*	I	G	R	I	**R**	P	L	A	E
Human *KDM5C*	I	A	K	I	**R**	P	I	A	E
Human *KDM5D*	I	A	K	I	**R**	P	I	A	E

Individual No. 7 **p.Ile465**

Human	Y	G	A	D	**I**	A	S	K	E
Rhesus	Y	G	A	D	**I**	A	S	K	E
Mouse	Y	G	A	D	**I**	A	S	K	E
Dog	Y	G	A	D	**I**	A	S	K	E
Elephant	Y	G	A	D	**I**	A	S	K	E
Chicken	Y	G	A	D	**I**	A	S	K	E
Xenopus	Y	G	A	D	**I**	A	S	K	E
Zebrafish	Y	G	A	D	**I**	A	S	K	E
Human *KDM5A*	Y	G	A	D	**I**	S	S	K	D
Human *KDM5C*	Y	G	A	D	**I**	H	S	K	E
Human *KDM5D*	Y	G	A	D	**I**	H	S	K	E

Individual No. 8 **p.Leu523**

Human	K	L	P	W	**L**	Y	V	G	M
Rhesus	K	L	P	W	**L**	Y	V	G	M
Mouse	K	L	P	W	**L**	Y	V	G	M
Dog	K	L	P	W	**L**	Y	V	G	M
Elephant	K	L	P	W	**L**	Y	V	G	M
Chicken	K	L	P	W	**L**	Y	V	G	M
Xenopus	K	L	P	W	**L**	Y	V	G	M
Zebrafish	T	L	P	W	**L**	Y	V	G	M
Human *KDM5A*	K	V	P	W	**L**	Y	V	G	M
Human *KDM5C*	K	V	P	W	**L**	Y	V	G	M
Human *KDM5D*	K	V	P	W	**L**	Y	V	G	M

Individual No. 13 **c.1026+2_1026+3del**

Human	T	N	A	G	T	G	A	G	T	G	A	C	A	T	C	T	C	C	C	T	T
Rhesus	T	N	A	G	T	G	A	G	T	G	A	C	A	T	C	T	C	C	C	T	T
Mouse	A	T	A	G	T	G	A	G	T	-	-	-	-	-	-	-	-	-	-	-	-
Dog	T	N	A	G	T	G	A	G	T	G	A	C	A	T	T	T	-	-	-	-	-
Elephant	T	S	A	G	T	G	A	G	T	G	A	C	A	C	C	T	-	-	-	-	-
Chicken	T	S	A	G	T	G	A	G	T	-	-	-	-	-	-	-	-	-	-	-	-
Xenopus	-	-	A	-	-	-	-	-	-	-	-	-	-	-	-	-	-	-	-	-	-
Zebrafish	-	-	A	-	-	-	-	-	-	-	-	-	-	-	-	-	-	-	-	-	-

Individual No. 14 **c.1464+3A>G**

Human	P	E	E	E	G	T	A	G	G	C
Rhesus	P	E	E	E	G	T	A	G	G	C
Mouse	P	E	E	E	G	T	A	G	G	C
Dog	P	E	E	E	G	T	A	G	G	C
Elephant	P	E	E	E	G	T	A	G	G	C
Chicken	P	E	E	E	G	T	G	G	G	-
Xenopus	P	E	D	E	G	T	T	A	G	-
Zebrafish	P	H	D	E	G	-	-	-	-	-

Individual No. 20 **p.Cys1231**

Human	Q	C	E	L	**C**	R	D	A	F
Rhesus	Q	C	E	L	**C**	R	D	A	F
Mouse	Q	C	E	L	**C**	R	D	A	F
Dog	Q	C	E	L	**C**	R	D	A	F
Elephant	Q	C	E	L	**C**	R	D	A	F
Chicken	Q	C	E	L	**C**	R	G	F	F
Xenopus	Q	C	E	L	**C**	R	S	C	F
Zebrafish	Q	C	E	L	**C**	R	D	A	F
Human *KDM5A*	Q	C	E	L	**C**	K	D	W	F
Human *KDM5C*	Q	C	D	L	**C**	Q	D	W	F
Human *KDM5D*	Q	C	D	L	**C**	Q	D	W	F

Individual No. 21 **p.Glu2**

Human		M		E	A		A		T	T
Rhesus		-		-	-		-		-	-
Mouse		M		E	P		A		T	T
Dog		-		-	-		-		-	-
Elephant		-		-	-		-		-	-
Chicken		A		P	P	G	G	-	-	-
Xenopus		-		-	-		-		-	-
Zebrafish	A	-	-	-	-	-	A	G	-	-
Human *KDM5A*		-		-	-		-		-	-
Human *KDM5C*		-		-	-		-		-	-
Human *KDM5D*		-		-	-		-		-	-

Table S1. Phenotypic characteristics of individuals with *KDM5B* variants

Individual No.	Sex	Age at Study	Height/Weight/HC	DD	ID	Autistic traits	Neurologic disorders or anomalies	Facial dysmorphism	Malformation and anomalies	Other medical issues
**1**	F	-	P3-P98<br>P3-P98<br>P3-P98	-	Severe	-	Behavioral problems (automutilation)	-	-	-
**2**	F	-	P3-P98<br>P3-P98<br>+ 2 SD	-	Normal IQ	-	-	Broad forehead<br>Synophrys<br>Epicanthal folds<br>Telecanthus<br>Small nose<br>Broad mouth	Joint hypermobility	Feeding difficulties<br>Hypotension<br>Skin abnormalities: keratosis pilaris, ulerythema ophryogenes
**3**	M	18	175.26 cm (P52)<br>112.5 Kg (>P99 +3 SD)<br>-	-	Moderate	-	Irritable, behavior problems with school<br>Anxiety	Macrostomia<br>Ear tag	Dental abnormalities (missing teeth)<br>Undescended testicle<br>brachycephaly	Obstructive sleep apnea<br>Frequent otitis media<br>Chronic constipation<br>Chronic kidney disease<br>Rhythmic movement disorder
**4**	M	3	103.2 cm (P97)<br>17.6 Kg (P96)<br>51 cm (P81)	Speech delay	-	Yes	-	-	Atrophic hands	-
**5**	M	10	150.8 (P97)<br>44.9kg (P94)<br>54cm (P67)	-	Normal IQ	-	ADHD<br>Graphomotor disorder	Prominent large ears<br>Low eyebrows<br>Telecanthus<br>Full nasal tip<br>Broad mouth	Mild joint hypermobility	Possible sleep apnea<br>Sleeping disorder<br>Convergence insufficiency<br>Multiple food allergies<br>Chronic skin rashes
**6**	F	2 DAYS	41 cm (<P1 -3.9 SD)<br>1.81 kg (<P1 -3.1 SD)<br>30 cm (<P1 -3.3 SD)	-	-	-	Left perisylvian Polymicrogyria<br>Enlarged extra-axial spaces which may represent micrencephaly<br>T1 prolongation in the brainstemh Hypothalamus, and thalami	Midface hypoplasia<br>Ear tags<br>Low set ears<br>Left ear: Grade I microtia, rudimentary ear, no opening of auditory meatus<br>Right ear: Auditory meatal opening but malformed, prominent skin tags associate<br>Microretrognathica	-	Right aortic arch with aberrant left subclavian artery and left ligamentum (vascular ring),<br>Muscular VSD, ASD, dilated/hypertensive R ventricule<br>Renal: Moderate left hydronephrosis and hydroureter
**7**	F	-	-	Global	Yes	Yes	Agenesis of corpus callossum<br>Septo-optic dysplasia	-	Hydrocephalus<br>Renal duplication	Hypertrichosis<br>Impaired pain sensation
**8**	F	13	107 cm (<1P, -7.1 SD)<br>121 kg (>99P, +5.4 SD)<br>52.5 cm (P20)	Non-verbal	Severe	Yes	Psychomotor slowing<br>Decreases spontaneity of behavior	Broad forehead<br>Large ears (mild)<br>Synophys<br>Low eyebrows<br>small nose,	Fingers with swan neck deformity<br>Atrophic hands	Obstructive sleep apnea<br>Recurrent pneumonias<br>Auditory processing

								Full nasal tip<br>Depressed nasal bridge		
**9**	F	14	175cm (P>99, +1.8 SD)<br>61.3 Kg (P79)<br>55 cm (P82)	Speech delay	Normal IQ	-	-	Midface retraction<br>Large ears<br>Low eyebrows<br>Full nasal tip	Atrophic hands<br>Fingers with swan neck deformity<br>And joint hypermobility	Sleeping disorder<br>Recurrent respiratory infections (otitis media)<br>Metabolic disorder (low guanidinoacetate)
**10**	M	15	167.6 cm (P40)<br>52.16 Kg (P31)<br>-	Delayed, difficult to understand<br>Auditory processing disorder	Moderate	-	Behavioral problems (Impatient, attention issues)<br>Seizures	Prominent forehead<br>Prominent<br>Slightly large ears<br>Down-slanting eyes<br>Slightly small philtrum	Dolichocephaly	Sleeping problems (snoring)<br>Pneumonia<br>Auditory issues
**11**	M	10	-	Gross and fine motors delays,<br>Auditory processing disorder	No	-	-	Maxillary hypoplasia<br>broad midportion<br>low bridge	Joint hypermobility, hydronephrosis with duplicated collecting system	Central apnea<br>Macrocephaly, abnormal muscle biopsy (congenital fiber type disproportion)
**12**	F	5	-	Global developmenta l delays	-	Yes	-	Hypertelorism,<br>Broad nasal root	Widden nipples	Feeding difficulty<br>Congenital hypothyroidism<br>Strabismus<br>Laryngeal cleft
**13**	M	11	-	-	-	-	-	-	Trigonocephaly<br>Metopic craniocynostosis<br>Hypospadias<br>Cryptorchidism<br>Abdominal wall hernia<br>Intestinal malrotation	-
**14**	F	4	104 cm (P77)<br>15.7 kg (P31)<br>49.6 cm (P50)	Development al delay with regression at age 2<br>Inability to sit or verbalize	Severe	Yes	Stereotypic hand movements, hypotonia; MRI showed delayed myelination	Epicanthal folds<br>Deep philtrum	-	Sleep disturbances
**15**	M	17	177cm (P70)<br>131.3kg (>P99 + 4.6 SD)<br>62.5cm (>P99 +5.2 SD)	Speech delays<br>Dysarthria	Mild	Yes	-	-	-	Cowden-like, with macrocephaly ganglioblastoma Vascular PHOST malformation
**16**	F	4	109.6 cm (P98)<br>8.90 kg (<P1 -4.2 SD)<br>50.5 cm (P72)	Gross motor delays<br>Speech delays	Normal IQ	Yes	Behavioral problems (Behavioral outbursts, anxiety)	-	-	Sleeping difficulties<br>Chronic constipation
**17**	M	8	138 cm (P96)<br>35.6 kg (P95)<br>54.5 cm (P95)	Delayed fine motor development	Normal IQ	Yes, Anxiety and phobias	Hyperextensible ADHD,	Midface hypoplasia<br>Synophrys	ASD repaired at 3<br>Inguinal hernia repair	Disrupted sleep

				Learning disabilities			Abnormal social interaction	over folded ear helices<br>Disordered hair patterning at vertex		
**18**	M	20	158.5 cm (P1 -2.5 SD)<br>97 kg (P96)<br>57 cm (P91)	Severe speech/language delay	Mild to moderate	Yes	ADHD	Low posterior hairline, narrow forehead, trigonocephaly, prominent supraorbital bridge, strabismus, flattened nasal tip, narrow nasal septum	Short tapered fingers (<3rd centile), short toes. Small penis	Sleep disturbance, Myopia
**19**	M	18	AT BIRTH<br>51 cm (P62)<br>3.3 kg (P18)<br>-<br><br>AT THE LAST EXAM<br>197.5 cm (P>99, +3.2 SD)<br>117.5 kg (P>99, +3.3 SD)<br>59 cm (P>99, +2.7 SD)	Delayed language development R/logopedics	Borderline (Total IQ 76, Verbal IQ 81, Performal IQ 74	Yes, also tantrums R/ Abilify	Hypotonia<br>Tantrums, migraine, moves rather slowly, developmental coordination disorder	Brachycephaly, long face, high forehead, temporally sparse hair, bushy eyebrows, synophrys, bilaterally temporal narrowing, downslanted palpebral fissures, short philtrum, facial asymmetry (left side slightly shorter), broad and long neck	Large hands, long, tapered fingers	Underwent correction of micrognathia with a bilateral sagittal split osteotomy of the mandibula and a bilateral Le Fort 1 osteotomy of the mandibula with extraction of third molars
**20**	F	28	162.5 cm (P45)<br>-<br>57 cm (P>99, +2.4 SD)	-	Mild	-	Behavioral problems (mood swings)	Midface retraction<br>Broad forehead<br>Large ears<br>Low eyebrows<br>Full nasal tip<br>Synophrys<br>Epicanthal folds<br>Telecanthus<br>Broad mouth<br>Depressed nasal bridge	Atrophic hands<br>Fingers with swan neck deformity<br>Joint hypermobility	
**21**	M	4Y4M	108.4 cm (P93)<br>19.6 kg (P91)<br>56 cm (>P99 +4.1 SD)	Gross motor delay<br>Speech delay	Mild	No	Meltdowns, aggressive, easily frustrated, problems with transitioning.<br><br>Decreased white matter with thinning of corpus callosum, enlarged lateral ventricles, mega cisterna magna, inferior vermian hypoplasia	Broad forehead<br>Midface retraction<br>Full nasal tip<br>Micrognathia	-	Hypospadias<br>Hyperopia

Height, Weight, and HC percentiles were calculated using https://simulconsult.com/resources/measurement.html?type=head

Table S2. *KDM5B* variants ACMG classification

Individual No.	Genetic variant (hg19/GRCh37)	cDNA variant (NM_001314042.1)	Protein variant	ACMG classification[1]	ACMG criteria used for classification
**1**	chr1:g.202702777A>G	c.3769T>C	p.Cys1257Arg	Likely pathogenic	PP3, PM2
**2**	chr1:g.202710661A>G	c.2887T>C	p.Cys963Arg	VUS	PM2
**3**	chr1:g.202718129C>T	c.2068G>A	p.Asp690Asn	VUS	PM2
**4**	chr1:g.202722085T>C	c.1757A>G	p.His586Arg	VUS	PP3, PM2
**5**	chr1:g.202715006G>A	c.2411C>T	p.Ala804Val	VUS	PP3, PM2
**6**	chr1:g.202777277G>A	c.157C>T	p.Arg53Trp	VUS	PM2, BP4
**7**	chr1:g.202725556A>C	c.1394T>G	p.Ile465Ser	VUS	PP3, PM2
**8**	chr1:g.202724476C>G	c.1569G>C	p.Leu523Phe	VUS	PP3, PM2
**9**	chr1:g.202719900G>A	c.1816C>T	p.Arg606Ter	Likely pathogenic	PVS1, PM2
**10**	chr1:g.202702711G>A	c.3835C>T	p.Arg1279Ter	Pathogenic	PVS1, PM2, PP5
**11**	chr1:g.202702711G>A	c.3835C>T	p.Arg1279Ter	Pathogenic	PVS1, PM2, PP5
**12**	chr1:g.202727602G>A	c.1222C>T	p.Gln408Ter	Likely pathogenic	PVS1, PM2
**13**	chr1:202731824:CACA:CA Deletion (2bp)a	c.1026+2_1026+3del	N/A	Likely pathogenic	PVS1, PM2
**14**	chr1:g.202725483T>C	c.1464+3A>G	N/A	VUS	PP3, PM2
**15**	chr1:g.202711876TTGTC>T	c.2485_2488del	p.Asp829MetfsTer9	Likely pathogenic	PVS1, PM2
**16**	chr1:g.202733231_202733240del	c.853_862del	p.Thr285GlufsTer5	Likely pathogenic	PVS1, PM2
**17**	chr1:g.202704625insA	c.3463_3464insT	p.Ser1155MetfsTer9	Likely pathogenic	PVS1, PM2
**18**	chr1 :g.202742411AACTdel	c.408_411del	p.Val137GlnfsTer54	Likely pathogenic	PVS1, PM2
**19**	chr1:g.202320001_203070000del	N/A	N/A		
**20**	chr1:g.202709936G>A	c.3058C>T	p.Arg1020Ter	Likely pathogenic	PVS1, PM2
	chr1:g.202702855A>G	c.3691T>C	p.Cys1231Arg	Likely pathogenic	PP3, PM2
**21**	chr1:g.202777430C>A	c.4G>T	p.Glu2Ter	Likely pathogenic	PVS1, PM2
	chr1:g.202729568C>T	c.1160G>A	p.Trp387Ter	Likely pathogenic	PVS1, PM2

[1] ACMG scores were retrieved from Franklin by genoox (https://franklin.genoox.com/clinical-db/home) and from Varsome (https://varsome.com)

Variant of uncertain significance (VUS), Pathogenic Supporting (PP), Pathogenic Moderate (PM), Pathogenic Very Strong (PVS), Benign Supporting (BP)

Table S3. Detailed explanation of the cases retained from the literature	
**Study**	**Details**
**Lebrun et al. (*Gene*, 2018)**	We excluded individual No. 1 since, as explained in the article, her sister presented with a similar phenotype but did not have a variant in *KDM5B*, suggesting that another gene variant could potentially explain the phenotype in the family. We decided to only include individuals with dominant *KDM5B* variants if these were de novo and not found in controls. For this reason, we also excluded certain individuals who had variants inherited from an asymptomatic parent: p.Arg733Gln, p.Ser828Tyr, p.Glu903Gln, p.Val1014Leu, and p.Arg1382Gln. Dominant variants c.576+2T>C and c.916+1G>A were retained.
**Lebon et al. (*Genes*, 2021)**	We excluded individual No. 2 due to the scarcity of phenotypic information. Variants p.Ala635Thr and p.Ser1155AlafsTer4 were retained.
**Martin et al. (*Science*, 2018)**	The following individuals were excluded: 202698996, 202705472, 202718254, 202733210. Dominant variants c.2697+3_2697+5del, p.Arg787Ter, p.Arg659Ter, p.Ile515Thr, and p.Arg1094SerfsTer5 were retained. The bi-allelic variants p.Leu1406Ter, c.2583-2A>G, p.Arg335Ter, p.Tyr208LeufsTer5, p.Asn1338ArgfsTer44, and c.1027-1G>A were retained.
**Mangano et al. (*Seizure*, 2022)**	The individual was excluded because phenotypic information could not be linked with certainty to the *KDM5B* variant since the individual also had two de novo variants and a 2q deletion of 8.2 Mb.
**Faundes et al. (*Am. J. Hum. Genet.*, 2018)**	Variants p.Leu1406Ter, c.2583-2A>G, p.Arg335Ter, p.Asn1338LysfsTer45, and p.Tyr208LeufsTer5 were retained.
